# Business Associates’ Involvement in US Health Care Data Breaches: Longitudinal Analysis

**DOI:** 10.2196/93588

**Published:** 2026-10-06

**Authors:** Martin Ignatovski

**Affiliations:** 1 Graduate School, Capitol Technology University, 11301 Springfield Rd, Laurel, MD, 20708, United States, 1 219 210 2108

**Keywords:** computer security, data security, health information management, Health Insurance Portability and Accountability Act, HIPAA, business associate, data breach, third-party risk, protected health information, cybersecurity, health care

## Abstract

**Background:**

Health care organizations increasingly rely on business associates (BAs) to provide clinical, administrative, and technology services that require access to protected health information. While the Health Information Technology for Economic and Clinical Health (HITECH) Act and the Health Insurance Portability and Accountability Act (HIPAA) Omnibus Rule extended legal liability to BAs, the frequency and characteristics of data breaches involving BAs have not been systematically tracked across the entire post-HITECH reporting era. Understanding these trends is critical for health information managers and cybersecurity professionals who are directly responsible for managing third-party risk.

**Objective:**

The author examined the longitudinal trends in BA involvement in health care data breaches reported to the US Department of Health and Human Services (HHS) Office for Civil Rights (OCR) from 2009 to 2025, including changes in frequency, breach mechanisms, breach locations, and severity profiles of BA-involved incidents across 3 regulatory periods.

**Methods:**

The author conducted a retrospective longitudinal analysis of health care data breaches (N=6612) reported to the HHS OCR breach portal between October 2009 and December 2025. The author operationalized BA involvement as breaches reported by BA entities or flagged as BA-related. Using logistic regression models, the author estimated annual trends in BA involvement, breach mechanism, and breach location. Chi-square tests assessed associations between BA status and breach characteristics across 3 regulatory periods: pre-Omnibus (2009‐2013), post-Omnibus (2014‐2019), and 2020‐2025. Proportion tests compared BA-involvement rates across periods.

**Results:**

BA-involved breaches accounted for 1950 of 6612 (29.5%) incidents and 285,718,494 (48.8%) of all affected individuals. The annual BA-involvement rate increased from 22.1% in the pre-Omnibus period to 36.6% in the 2020‐2025 period (*z* score=8.29, *P*<.001). Logistic regression confirmed an 8% annual increase in the odds of BA involvement (odds ratio [OR] 1.08, 95% CI 1.07‐1.10; *P*<.001). Hacking/IT incidents shifted from a minority of incidents to the dominant breach mechanism (OR 1.41 per year, 95% CI 1.39‐1.44; *P*<.001), and the odds of network server breaches increased by 29% per year (OR 1.29, 95% CI 1.26‐1.31; *P*<.001). BA-involved breaches were significantly more concentrated in hacking (1282/1950, 65.7% vs 2351/4662, 50.4%) and network server locations (1084/1950, 55.6% vs 1435/4662, 30.8%) compared with non-BA breaches (*P*<.001). The proportion of mega breaches (≥100,000 individuals) also increased annually (OR 1.16, 95% CI 1.13‐1.19; *P*<.001), with BA-involved breaches exhibiting a significantly higher rate of mega breaches (12.4% vs 8.2%; *χ*^2^_1_=28.44; *P*<.001).

**Conclusions:**

Building on prior evidence linking BA involvement to breach severity, this study demonstrates that BA-involved health care data breaches accelerated substantially across the post-HITECH reporting era, with the steepest increase beginning in 2020. The concurrent growth of hacking and the concentration of breaches on network servers coincided with digital transformation, cloud migration, and the ransomware epidemic, which may have amplified third-party risk exposure. Health information managers and cybersecurity professionals should prioritize BA risk management strategies that account for the evolving threat landscape, including enhanced vendor security assessments and data compartmentalization requirements.

## Introduction

The number of data breaches in the US health care industry has increased over the last 16 years, endangering the confidentiality, availability, and integrity of protected health information (PHI). The Health Insurance Portability and Accountability Act (HIPAA) mandates that health care organizations notify the Office for Civil Rights (OCR) of the US Department of Health and Human Services (HHS) of any data breaches impacting 500 or more individuals [1,2]. OCR’s reporting website, which covers the period from October 2009 through the end of 2025, is the most extensive longitudinal record of health care data breaches in the United States [3,4].

Business associates (BAs), which include third-party vendors, contractors, and service providers that store, maintain, or transmit PHI on behalf of covered entities (CEs), account for a significant percentage of health care data breaches [5]. BAs include third parties that provide cloud hosting, billing and coding, data analytics, health information exchange, and IT infrastructure management [6,7]. While the Health Information Technology for Economic and Clinical Health (HITECH) Act of 2009 initially established breach notification requirements and reinforced HIPAA enforcement, the HIPAA Omnibus Rule of 2013 extended direct regulatory liability and the HIPAA Security Rule’s requirements to BAs [8,9]. The goal of these regulatory actions was to address the growing threat posed by third-party relationships to the security and privacy of PHI.

The number of electronic PHI records handled by BAs on behalf of health care organizations increased in parallel with the digitization of health records and HITECH’s meaningful use incentives [10]. CEs outsourced significant data processing to outside providers when clinical and administrative systems were moved to cloud-based platforms [11]. The digital transformation in health care, accelerated by the COVID-19 pandemic, prompted the adoption of third-party data processing services, telehealth and telemedicine platforms, and remote collaboration tools [12,13]. These developments coincided with an increase in the percentage of PHI under BA stewardship and a broader attack surface for malicious actors.

The rise of third-party data handling changed the cyberthreat landscape. After 2019, ransomware attacks on health care organizations rose sharply. Sophisticated threat actors specifically targeted network servers and cloud infrastructure, exploiting supply chain weaknesses to exfiltrate large volumes of data [14,15]. BAs became prime targets because a compromised BA with access to a network server or cloud infrastructure can expose PHI from multiple CEs simultaneously [16].

Multiple recent studies on health care data breaches investigated the causes of breaches [17-19], the influence of entity type on breach severity [12,19], the correlation between breach types and organizational characteristics [20], and strategies for prevention [21,22]. Notably, prior work, including a study by the present author, has established that BA involvement is associated with both breach severity and the number of impacted individuals [12]. However, the current research literature does not provide comprehensive longitudinal monitoring of BA involvement across the entire post-HITECH reporting period, nor does it analyze how the characteristics of BA-related breaches, including breach mechanisms, locations, and severity profiles, evolved across distinct regulatory eras. These trends are important for cybersecurity specialists and health information managers who must implement risk management strategies to mitigate the impact of third-party attacks.

The author of this study addressed two research questions: (1) What is the trend in BA involvement in reported US health care data breaches from 2009 to 2025, and are there significant differences across regulatory periods? (2) How do the breach mechanism, breach location, and severity characteristics of BA-involved breaches evolve over the same period? The author used the entire dataset of breaches reported to the HHS OCR breach portal to conduct an exhaustive longitudinal analysis of third-party risk trends in the US health care system.

## Methods

### Study Design

This research used a retrospective longitudinal design, drawing on archival secondary data from the HHS OCR breach portal. The analysis included all reported health care data breaches from October 21, 2009, to December 30, 2025. The study used publicly accessible, deidentified archival data without involving human participants.

### Data Source and Extraction

The author extracted the dataset from the HHS OCR breach portal, which tracks all HIPAA breach reports affecting 500 or more individuals [3]. The portal is a publicly accessible, government-maintained data source. Many earlier studies on health care data breaches used the same data source [4,12,17,20,23]. The author used a five-step process to ensure data accuracy: (1) identifying and confirming the data source; (2) extracting and storing the full dataset; (3) checking and filtering the data for quality, including removing 1 record that was missing a value for the number of impacted individuals; (4) removing duplicate entries based on matching entity name, reporting date, and number of impacted individuals; and (5) loading the final dataset into statistical software for analysis.

The initial data extract contained 6613 breach reports. One record was missing a value for the number of impacted individuals and was removed because this variable is required for the severity, concentration, and log-severity analyses. The final analytic dataset included 6612 breach reports. Each breach record included the name of the reporting entity, the state, the entity type (health care provider, health plan, health care clearinghouse, or BA), the number of impacted individuals, the date of the breach submission, the type of breach, the location of the breached information, a flag for BA involvement, and a web description field. The most recent breach in the dataset at the time of extraction was reported on December 30, 2025, which defines the end of the study window. Because breach notifications are subject to reporting and public-posting lags, data for 2025 were incomplete as of this date and likely understate the true incident count for that year. All 6612 breaches in the analytic dataset were retained in the analysis; 2025 figures should be interpreted as provisional.

### Variable Definitions

The author identified *BA involvement* as the primary independent variable, which was a binary indicator set to 1 when the reporting entity was classified as a BA or when the *BA present* field was set to “Yes.” This dual operationalization encompassed both direct breaches reported by BAs and breaches reported by CEs that identified BA involvement. Using this definition, the author classified 1950 of the 6612 (29.5%) breaches as BA-involved.

The author classified the *breach type* variable into 6 groups based on HHS classification: hacking/IT incident, unauthorized access/disclosure, theft, loss, improper disposal, and other/multiple. Similarly, the author used the HHS classification to categorize the *breach location* variable into 7 categories: network server, email, paper/films, laptop, electronic medical record (EMR), desktop computer, and other. The author used the *number of impacted individuals* as the measure of breach severity and treated it as a continuous variable. Finally, the author created a binary *mega breach* variable defined as 100,000 or more impacted individuals.

To analyze temporal patterns across regulatory milestones, the author categorized breaches into 3 periods: pre-Omnibus (2009‐2013, n=911), post-Omnibus (2014‐2019, n=2150), and 2020‐2025 (n=3551). Although the HIPAA Omnibus Rule was published in January 2013, its compliance deadline was September 23, 2013. For most of 2013, CEs and BAs therefore remained subject to pre-Omnibus requirements, and 2013 was accordingly grouped with the pre-Omnibus period. The author defined the period beginning in 2020 separately because it coincided with the COVID-19 pandemic, the ransomware epidemic, and accelerating cloud migration.

### Statistical Analysis

The author calculated descriptive statistics for all variables: frequencies, proportions, means, SDs, medians, and IQRs. The Shapiro-Wilk test indicated a significant departure from normality in the distribution of impacted individuals (*W*=0.04; *P*<.001). The distribution was right-skewed, with the mean exceeding the median (96,461 vs 3790) and a skewness coefficient of 57.16, supporting the use of nonparametric tests for bivariate comparisons. Because of this skew, the author used the Spearman rank-order correlation to assess the monotonic association between calendar year and the number of impacted individuals. A natural log transformation was applied to the number of impacted individuals for parametric severity modeling.

The author used logistic regression models to estimate yearly trends in BA involvement, hacking proportion, network server proportion, theft proportion, and mega breach probability, with calendar year as a continuous predictor variable. The results are reported as odds ratios (ORs) with 95% CIs. To estimate the annual trend in breach severity, the author fit an ordinary least squares regression model with the natural log of the number of impacted individuals as the dependent variable and calendar year as the predictor, reporting heteroskedasticity-consistent (HC3) robust SEs. The log transformation addressed the skewness of the severity distribution. Additionally, the author used 2-proportion *z*-tests to compare BA-involvement rates across regulatory periods.

Chi-square tests of independence evaluated the relationships between BA involvement and breach type, BA involvement and breach location, breach type and mega breach status, and breach location and mega-breach status. The author calculated Cramér *V* to quantify effect size. Kruskal-Wallis tests evaluated the distributions of impacted individuals by breach type and location, with pairwise Mann-Whitney *U* tests used for post hoc comparisons. All tests were 2-tailed, and the significance level was set at *P*<.05.

To characterize the concentration of breach impact, the author computed the Gini coefficient for the number of impacted individuals, using the individual breach incident as the unit of analysis. The coefficient was derived from the Lorenz curve as the ratio of the area between the line of perfect equality and the observed Lorenz curve to the total area beneath the line of equality, ranging from 0, where all breaches impact an equal number of individuals, to 1, where the entire impact is concentrated in a single breach. Concentration was also summarized as the share of all impacted individuals accounted for by the top 1% (67/6612) of breaches ranked by severity. These measures describe how the total population of impacted individuals was distributed across the 6612 breaches, rather than across entities or reporting years.

The author used statsmodels (version 0.14), SciPy (version 1.12), and pandas (version 2.2) to analyze the data in Python 3.12.

### Ethical Considerations

The author analyzed publicly available, deidentified archival data from a US government agency. No individual patient data were accessed or analyzed. The HHS OCR breach portal contains only organizational-level information on breach reports. Accordingly, this research did not involve human participants and did not require institutional review board approval.

## Results

### Overview of Reported Breaches

The HHS OCR received reports of 6612 health care data breaches that impacted 500 or more individuals between October 2009 and December 2025. All these breaches impacted a total of 637,803,323 individuals. From 199 breaches in 2010 to a peak of 741 in 2023, the number of breaches reported each year rose substantially during the study period. Health care providers accounted for the majority of reported breaches (n=4758, 72.0%). BAs followed (n=993, 15.0%), then health plans (n=841, 12.7%), and health care clearinghouses (n=15, 0.2%). Entity type was not recorded for the remaining 5 breaches. Hacking/IT incidents were the most common breach type (n=3633, 54.9%), followed by unauthorized access/disclosure (n=1483, 22.4%), theft (n=996, 15.1%), loss (n=214, 3.2%), improper disposal (n=114, 1.7%), and other/multiple types (n=172, 2.6%). Network servers were the most frequent breach location (n=2519, 38.1%), followed by email (n=1460, 22.1%) and paper/films (n=864, 13.1%).

### BA-Involvement Rates Across Regulatory Periods

Of the 6612 reported data breaches, 1950 (29.5%) involved a BA. BA-involved breaches accounted for 285,718,494 of the total 637,803,323 (44.8%) impacted individuals. The annual BA-involvement rate showed an upward trajectory across the study period (Table 1). In the pre-Omnibus period (2009‐2013), BA-involved breaches accounted for 22.1% of reported data breaches. During the post-Omnibus period (2014‐2019), the rate remained largely stable at 20.9%. A substantial increase occurred in the 2020‐2025 period, during which BA-involved breaches accounted for 36.6% of reported breaches.

**Table 1.** Annual trends in health care data breaches and business associate (BA) involvement, 2009‐2025.

Year	Total breaches	BA-involved^a^, n/N	BA rate (%)	Mega breaches^b^, n	Mega rate (%)	Hacking, n/N (%)
2009^c^	18	3/18	16.7	0	0.0	0/18 (0.0)
2010	199	46/199	23.1	10	5.0	8/199 (4.0)
2011	200	45/200	22.5	10	5.0	15/200 (7.5)
2012	218	40/218	18.3	5	2.3	10/218 (4.6)
2013	276	67/276	24.3	6	2.2	27/276 (9.8)
2014	314	81/314	25.8	11	3.5	35/314 (11.1)
2015	270	45/270	16.7	12	4.4	56/270 (20.7)
2016	328	68/328	20.7	14	4.3	114/328 (34.8)
2017	358	46/358	12.8	9	2.5	149/358 (41.6)
2018	369	90/369	24.4	23	6.2	165/369 (44.7)
2019	511	119/511	23.3	42	8.2	314/511 (61.4)
2020	663	264/663	39.8	75	11.3	457/663 (68.9)
2021	715	253/715	35.4	86	12.0	546/715 (76.4)
2022	719	254/719	35.3	89	12.4	569/719 (79.1)
2023	741	278/741	37.5	150	20.2	603/741 (81.4)
2024	535	174/535	32.5	68	12.7	432/535 (80.7)
2025^d^	178	77/178	43.3	13	7.3	133/178 (74.7)

a BA involvement was identified as breaches in which the reporting entity was classified as a business associate or the business associate present field was marked “Yes.”

b Mega breach is defined as ≥100,000 impacted individuals.

c 2009 includes only breaches reported from October 21.

d 2025 data are provisional and incomplete owing to reporting and posting lags.

Logistic regression with year as a continuous predictor confirmed a statistically significant annual increase in BA involvement (OR 1.08, 95% CI 1.07‐1.10; *P*<.001), indicating that the odds of BA involvement increased by approximately 8% per year. The model-predicted probability of BA involvement rose from 17.0% (95% CI 15.1‐19.1) in 2010 to 39.6% (95% CI 37.4‐41.8) in 2025, an increase of 22.5 percentage points across the study period (Figure 1). Two-proportion *z*-tests revealed a significant difference between the pre-Omnibus and 2020‐2025 BA-involvement rates (22.1% vs 36.6%; *z* score=8.29; *P*<.001). The chi-square test for 3-period comparison was also significant (*χ*²_2_=187.30; *P*<.001), confirming that BA-involvement rates varied across regulatory periods (Table 1).

To assess whether the choice of regulatory-period boundaries influenced these results, the author conducted a sensitivity analysis varying the placement of 2013, the year the Omnibus Rule was published. Reassigning 2013 to the post-Omnibus period yielded pre-Omnibus and post-Omnibus BA-involvement rates of 21.1% and 21.3%, respectively, whereas excluding 2013 as a transitional year yielded rates of 21.1% and 20.9%, respectively. Under both alternatives, the 2020‐2025 rate remained unchanged at 36.6%, and the annual trend estimate was stable (OR 1.08, 95% CI 1.07-1.10 with 2013 included; OR 1.085, 95% CI 1.069-1.101 with 2013 excluded). The pattern of flat pre- and post-Omnibus rates followed by a marked 2020‐2025 increase was therefore robust to the categorization of 2013.

Because the 2025 data are provisional and incomplete, the author repeated the principal trend analysis excluding that year. The annual trend estimate was essentially identical (OR 1.08, 95% CI 1.06‐1.09; *P*<.001), and the BA-involvement rate for the 2020‐2024 period was 36.3% compared with 36.6% when 2025 was included. Therefore, the principal findings and conclusions do not depend on the inclusion of the provisional 2025 data.

**Figure 1.**
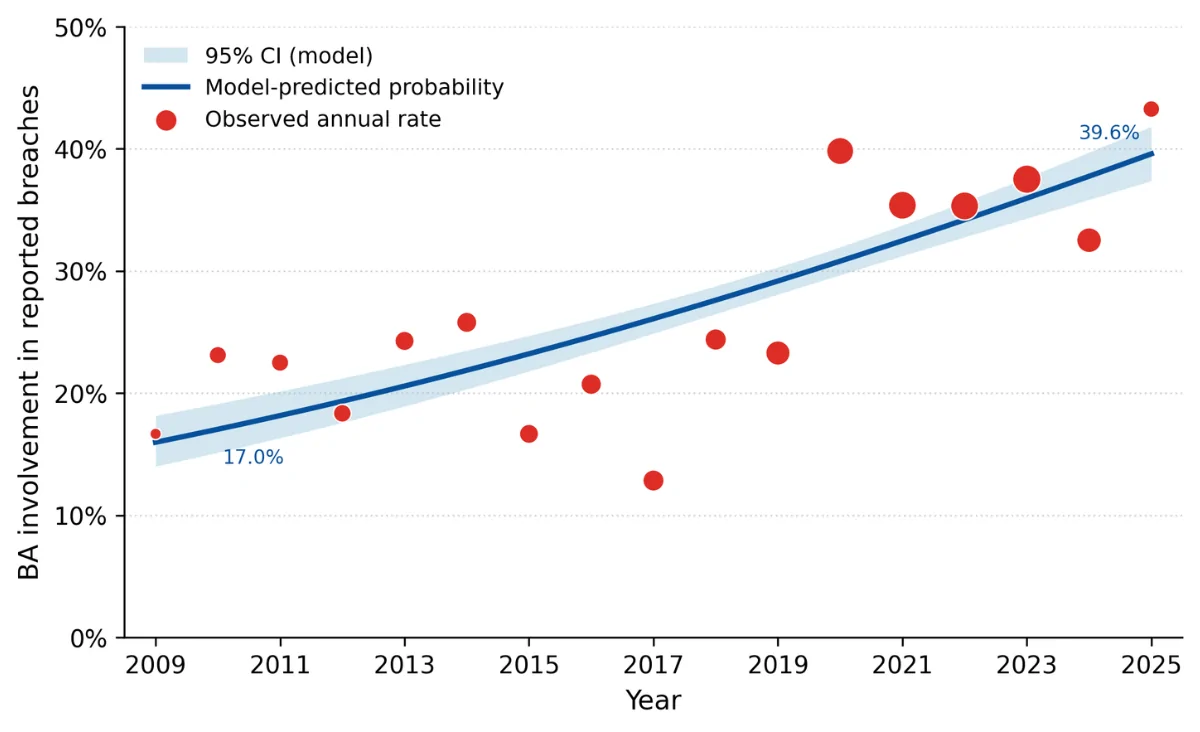
Model-predicted probability of business associate (BA) involvement in reported health care data breaches, United States, 2009‐2025. The solid line shows the predicted probability from a logistic regression with year as a continuous predictor; the shaded band is the 95% CI. Markers show observed annual rates, sized by the number of reported breaches that year. The 2025 data are provisional owing to reporting lags.

### Trends in Breach Mechanisms

The types of breaches changed significantly over the study period. Hacking/IT incidents, which accounted for a small minority of breaches early in the period, became the most frequently reported breach type. Logistic regression estimated that the odds of a breach being classified as a hacking/IT incident increased by 41% per year (OR 1.41, 95% CI 1.39‐1.44; *P*<.001). At the same time, theft-related breaches decreased significantly, from 67.8% (135/199) of the breaches in 2010 to under 2% (10/535) in 2024 (OR 0.69 per year, 95% CI 0.67‐0.70; *P*<.001; Table 2). These opposing trends, with hacking rising as theft declined, reflect the migration of breach risk from physical media to networked systems. A concurrent cross-sectional analysis of OCR breach data from 2010 to 2024 similarly documented the rise of hacking/IT incidents from 4% to 81% of reported breaches, with ransomware increasing as a subset before declining to 11% of breaches by 2024 [24]. BA-involved breaches were disproportionately concentrated in hacking mechanisms. Among BA-involved breaches, 65.7% (1282/1950) were classified as hacking/IT incidents, compared with 50.4% (2351/4662) among non-BA breaches. The chi-square test confirmed a significant association between BA status and breach type (*χ*²_5_=165.12, Cramér *V*=0.158; *P*<.001; Table 3).

**Table 2.** Logistic and linear regression models for annual trends, 2009‐2025.

Outcome	OR^a^ (95% CI)	*P* value	β^b^ (95% CI)	*P* value
BA^c^ involvement	1.08 (1.07‐1.10)	<.001	—^d^	—
Hacking	1.41 (1.39‐1.44)	<.001	—	—
Network server	1.29 (1.26‐1.31)	<.001	—	—
Mega breach	1.16 (1.13‐1.19)	<.001	—	—
Theft	0.69 (0.67‐0.70)	<.001	—	—
Log (severity)^e^	—	—	0.10 (0.09‐0.11)	<.001

a OR: odds ratio from logistic regression with year as continuous predictor.

b β: coefficient from ordinary least squares regression with robust standard errors (HC3).

c BA: business associate.

d Not applicable; binary outcomes were modeled with logistic regression (OR reported) and log(severity) with ordinary least squares regression (β reported).

e The log(Severity) model uses the natural logarithm of the number of impacted individuals as the dependent variable.

**Table 3.** Breach characteristics by business associate (BA) involvement status^a^.

Characteristic	BA-involved (n=1950)	Non-BA (n=4662)	Total (N=6612)	Test statistic (chi-square or Mann-Whitney *U* test)	*P* value
Breach type, n (%)					
Hacking/IT incident	1282 (65.7)	2351 (50.4)	3633 (54.9)	—^b^	—
Unauthorized access	390 (20.0)	1093 (23.4)	1483 (22.4)	—	—
Theft	164 (8.4)	832 (17.8)	996 (15.1)	165.12 (5)^c^	<.001
Loss	42 (2.2)	172 (3.7)	214 (3.2)	—	—
Improper disposal	20 (1.0)	94 (2.0)	114 (1.7)	—	—
Other or multiple	52 (2.7)	120 (2.6)	172 (2.6)	—	—
Breach location, n (%)					
Network server	1084 (55.6)	1435 (30.8)	2519 (38.1)	—	—
Email	263 (13.5)	1197 (25.7)	1460 (22.1)	—	—
Paper or films	243 (12.5)	621 (13.3)	864 (13.1)	402.64 (6)^c^	<.001
Laptop	62 (3.2)	313 (6.7)	375 (5.7)	—	—
EMR^d^	59 (3.0)	213 (4.6)	272 (4.1)	—	—
Desktop	21 (1.1)	175 (3.8)	196 (3.0)	—	—
Other/multiple	218 (11.2)	708 (15.2)	926 (14.0)	—	—
Severity					
Mean (SD)	146,522 (785,392)	75,522 (1,205,673)	96,461 (1,098,988)	—	—
Median (IQR)	4483 (1390-25,866)	3534 (1198-15,320)	3790 (1250-17,817)	4,915,792^e^	<.001
Mega breach, n (%)	242 (12.4)	381 (8.2)	623 (9.4)	28.44 (1)^c^	<.001

a Chi-square tests were used for categorical comparisons; Mann-Whitney *U* test was used for severity distributions. Mega breach was defined as ≥100,000 individuals.

b Not applicable.

c Chi-square test.

d EMR: electronic medical record.

e Mann-Whitney *U* test.

### Trends in Breach Locations

Network server concentration increased significantly over the study period. Logistic regression estimated a 29% annual increase in the odds of a breach occurring on a network server (OR 1.29, 95% CI 1.26‐1.31; *P*<.001). BA-involved breaches exhibited a significantly higher concentration on network servers: 55.6% (1084/1950) of the BA-involved breaches occurred on network servers, compared with 30.8% (1435/4662) of non-BA breaches. The association was significant (*χ*²_6_=402.64, Cramér V=0.247; *P*<.001), representing the strongest bivariate association identified in the analysis. Additionally, BA-involved breaches were less concentrated in email (263/1950, 13.5% vs 1197/4662, 25.7%), paper/films (243/1950, 12.5% vs 621/4662, 13.3%), and laptop locations (62/1950, 3.2% vs 313/4662, 6.7%). The Kruskal-Wallis test showed that breach severity differed significantly across breach locations (*H*=858.93; *P*<.001), with network server breaches exhibiting the highest median severity.

### Severity Trends and Mega Breaches

The impact of data breaches increased over the study period. A Spearman correlation between year and the number of impacted individuals was significant (ρ=0.21; *P*<.001). An ordinary least squares regression of log-transformed breach severity on year confirmed this trend, with each additional year associated with a 0.10 increase in log-transformed impacted individuals (*β*=.10, 95% CI 0.09‐0.11; *P*<.001). Because the model is log-linear, this coefficient corresponds to an approximate multiplicative change of exp(0.10), or roughly an 11% increase in the number of individuals impacted per breach per year. Logistic regression estimated a 16% annual increase in the odds of a mega breach (OR 1.16; *P*<.001). Of the 6612 breaches, 623 (9.4%) met the mega breach threshold of 100,000 or more impacted individuals (Tables 1 and 3).

BA-involved breaches demonstrated a significantly higher mega breach rate (242/1950, 12.4%) than non-BA breaches (381/4662, 8.2%; *χ*^2^_1_=28.44; *P*<.001). The Mann-Whitney *U* test confirmed that the overall severity distribution of BA-involved breaches was significantly higher than that of non-BA breaches (*U*=4,915,792; *P*<.001). BA-involved breaches had a mean of 146,522 (SD 785,392) impacted individuals compared with 75,522 (SD 1,205,673) for non-BA breaches. The median values were closer between the two: 4483 (IQR 1390‐25,866) for BA-involved versus 3534 (IQR 1198‐15,320) for non-BA breaches, suggesting that the mean difference was driven by a higher frequency of extreme-severity events among BA-involved incidents (Table 3).

### Concentration of Impact

The distribution of breach impact was highly concentrated. The Gini coefficient for impacted individuals across all breaches was 0.926, indicating that a small number of high-severity breaches accounted for most impacted individuals (Figure 2). The top 1% (n=67 incidents) of data breaches accounted for 55.5% (353,987,192/637,803,323) of all impacted individuals. Among these top 1% (67/6612) data breaches, 43 of 67 (64.2%) involved a BA. The BA share of total impacted individuals varied substantially by year, from a low of 4.8% (5,434,492/112,466,720) in 2015 to peaks exceeding 60% during the 2020‐2025 period (23,390,838/35,321,223, 63.4% in 2020 and 118,589,395/183,006,103, 64.8% in 2023), reflecting the outsized influence of individual catastrophic events on annual totals.

**Figure 2.**
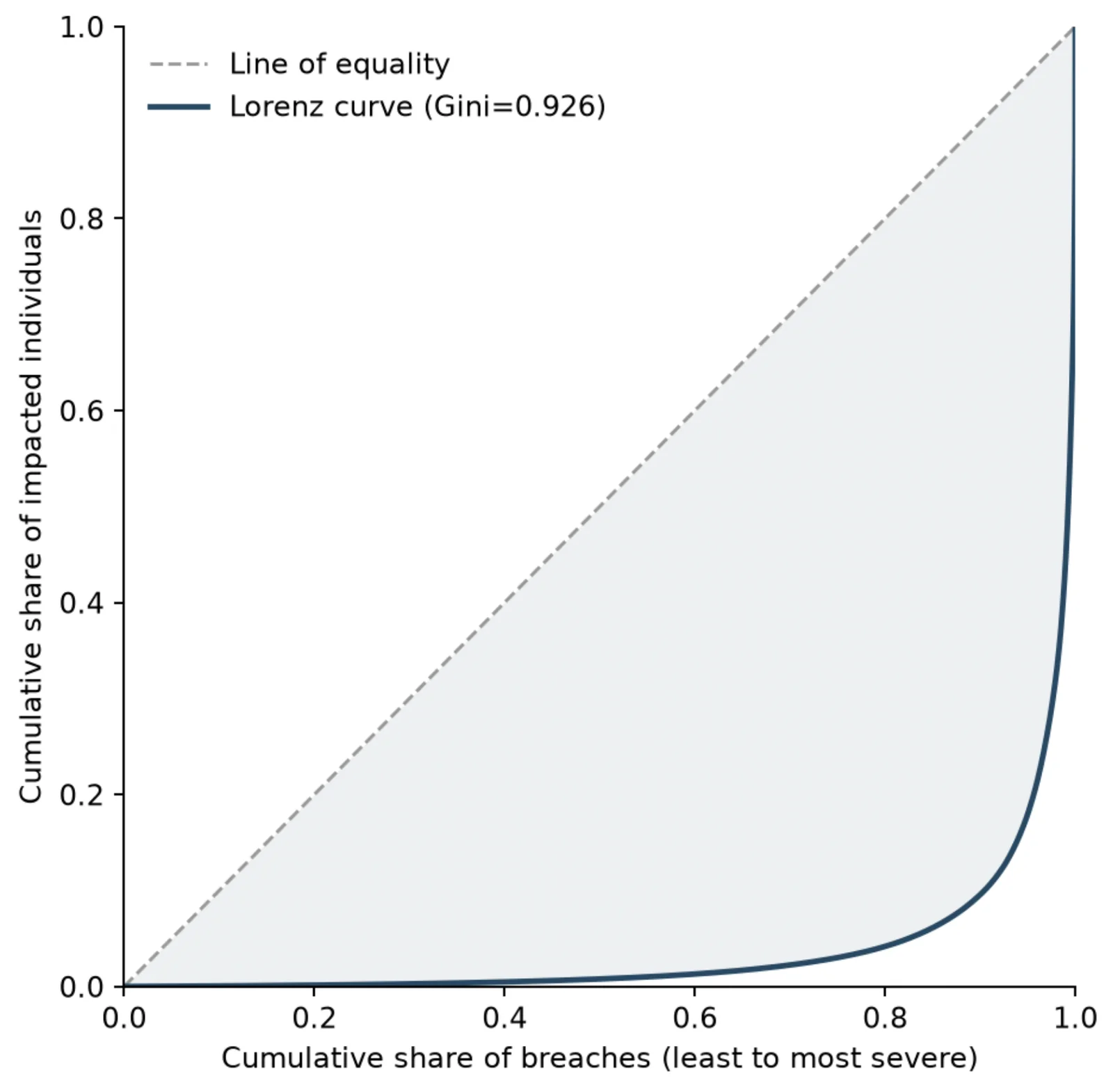
Lorenz curve of the distribution of impacted individuals across reported health care data breaches in the United States, 2009‐2025. The dashed diagonal represents perfect equality, where every breach impacts an identical number of individuals. The solid curve shows the observed cumulative distribution; its departure from the diagonal reflects the concentration of impact, with a Gini coefficient of 0.926. Breaches are ordered from least to most severe along the horizontal axis.

## Discussion

### Principal Findings

The author presents a longitudinal analysis of BA involvement in US health care data breaches over 16 years. The principal findings indicate that health care data breaches involving third-party BAs increased significantly, with the largest increase during the 2020‐2025 period. The rate of data breaches involving BAs grew from 22.1% in the pre-Omnibus period to 36.6% in the 2020‐2025 period, representing an increase of approximately 8% annually in the odds of BA involvement. Similarly, data breaches involving BAs were increasingly concentrated in hacking mechanisms and network server locations, mirroring broader changes in the health care cyberthreat landscape. These analyses establish the temporal trends and associations described above. Any attribution of these trends to specific external factors is interpretive and has not been formally tested.

The finding that BA-involvement rates remained flat between the pre-Omnibus and post-Omnibus periods (22.1% vs 20.9%) suggests that the regulatory extension of liability to BAs under the 2013 Omnibus Rule did not produce an immediate, detectable reduction in third-party breach frequency. This observation aligns with the existing literature, which highlights difficulties with HIPAA compliance within health care organizations and their BAs [5,7,9]. The marked increase in BA-involvement rates beginning in 2020 coincided with the COVID-19 pandemic, accelerated cloud migration, and a rise in ransomware attacks targeting health care infrastructure. While these concurrent developments offer a plausible explanation for the 2020‐2025 acceleration, the present analysis cannot establish a causal relationship, and this interpretation should be regarded as provisional.

### Interpreting BA Involvement

A central interpretive consideration concerns what the BA-involvement measure captures. Under the HIPAA Breach Notification Rule, a BA that discovers a breach must notify the affected CE. At the same time, the CE bears the ultimate responsibility for notifying impacted individuals and the Secretary of HHS (45 CFR 164.404, 164.408, and 164.410). The reporting entity recorded in the HHS data, therefore, reflects statutory and contractual notification arrangements rather than causal responsibility for the incident. In the present dataset, of the 1950 BA-involved breaches, 985 were classified under the BA entity type and were flagged as involving a BA, 957 were reported by a CE that identified BA involvement, and 8 were classified under the BA entity type without a separate involvement flag. The 985 and the 8 together constitute the 993 breaches for which the reporting entity’s type was recorded as a BA. A BA may thus be recorded as involved whether the breach originated within its systems, occurred in data it maintained, or was reported by a CE that flagged third-party involvement in the incident chain.

These 2 reporting pathways do not represent distinct categories of event but rather 2 administrative routes through which the same underlying phenomenon, the involvement of a BA in a reported breach, reaches the federal record. Whether a CE files and identifies a BA or the BA files directly, a third party was involved in the incident; what differs is the filing party determined by contractual and statutory notification arrangements, not whether BA involvement occurred. Combining the 2 operationalizes the intended construct, third-party involvement in a breach, rather than conflating separate event types, whereas separating them would instead measure which entity submitted the notification, a distinct question from whether a BA was involved. Accordingly, the measure should be interpreted as a third-party association with a reported breach rather than as an assignment of fault to the BA, and the attribution of risk directly to these entities should be made with corresponding caution.

The higher proportion of mega breaches among BA-involved incidents also warrants careful interpretation. Because a single BA frequently maintains PHI on behalf of many CEs, a compromise at 1 vendor can simultaneously expose records aggregated from numerous organizations. The elevated mega breach rate among BA-involved incidents may therefore reflect, in part, this data aggregation rather than a difference in the underlying security posture of BAs relative to CEs. This aggregation effect does not diminish the practical significance of the finding. On the contrary, it is precisely the concentration of multiple organizations’ data within individual third parties that makes BA relationships a critical locus of systemic risk, since the impact of a single vendor compromise can cascade across the many CEs it serves. A recent incident-level analysis of ransomware breaches reached a convergent conclusion, finding that BA-involved incidents were smaller on average yet disproportionately represented among the very largest breaches, consistent with a hub-and-spoke concentration of third-party risk [25].

Finally, the observed annual increase in the odds of BA involvement should be understood in the context of the expanding role of third parties in health care data processing over the study period. As CEs migrated clinical and administrative functions to cloud platforms and specialized vendors, the volume of PHI maintained by BAs grew substantially. The rising rate of BA involvement may, therefore, partly reflect this growth in the quantity of data under third-party stewardship rather than a change in per-unit risk alone. The present analysis could not separate these mechanisms because the HHS data do not include measures of data volume, organizational size, revenue, or specific security controls. The regression models accordingly estimate temporal associations without adjustment for these factors, and the trends should be interpreted as descriptive of the reported breach landscape rather than as evidence of a causal change in BA risk.

### Comparison With Prior Work

This study’s findings build on previous research on health care data breaches in several significant ways. Prior examinations of the HHS OCR breach portal have recorded the overall rise in breach frequency and the increasing prevalence of hacking as a breach method [17,18,26]. The current study introduces a systematic BA-specific perspective, illustrating that third-party involvement has increased disproportionately compared with overall breach trends. Previous research by the author revealed a substantial impact of entity type on the number of impacted individuals in COVID-era breaches [12] and documented correlations between breach types and organizational characteristics [19,20]. The present study does not attempt to re-establish that BAs are associated with severe breaches, which prior work has already shown; rather, its novel contribution is to track how that third-party risk has evolved continuously across 16 years and 3 distinct regulatory eras, demonstrating that the BA-severity differential endures and that BA-involved breaches have shifted increasingly toward high-severity hacking and network server vectors.

The concentration analysis reveals a critical dimension of third-party risk that descriptive breach counts do not capture. While BA-involved breaches accounted for 29.5% (1950/6612) of all reported incidents, they were responsible for 44.8% (285,718,494/637,803,323) of all impacted individuals and 64% (43/67) of the top 1% (67/6612) of breaches by severity. This asymmetrical concentration has important implications for risk assessment: the aggregate population impact of BA-involved breaches is substantially larger than the incident count alone would suggest.

### Practical Implications

The results of this study have many implications for professionals in health information management and cybersecurity. First, the growing share of breaches involving BAs underscores the importance of strong vendor risk management programs. The HIPAA Security Rule requires CEs to obtain satisfactory assurances from BAs regarding the security and privacy of PHI. However, the rule’s vague requirements give each organization considerable freedom [9]. Health information and cybersecurity experts should establish structured vendor security assessment programs that compare BA controls against well-known frameworks such as the HITRUST Common Security Framework or the National Institute of Standards and Technology Cybersecurity Framework [27,28].

Second, the disproportionate concentration of BA-involved breaches on network servers highlights the importance of data compartmentalization. Organizations should evaluate whether the volume of PHI accessible through third-party network connections is proportionate to the services provided by the BA. Limiting the scope of PHI accessible to BAs, implementing network segmentation, and requiring encryption at rest and in transit can reduce the potential impact of a third-party compromise [15].

Third, the finding that 64% (43/67) of the most serious breaches involved a BA indicates that organizational disaster recovery and incident response plans should clearly address situations involving a third party. Many CEs may not be able to assess the security of their BAs, making it harder to detect and respond to breaches [16].

### Regulatory and Policy Implications

The finding that a measurable change did not follow the 2013 Omnibus Rule in the rate of BA involvement carries implications for health care data protection policy. The absence of a measurable change in BA involvement around the 2013 Omnibus Rule was robust to the choice of transition-year classification in the sensitivity analysis, strengthening the inference that the extension of direct liability was not, by itself, associated with a detectable reduction in third-party breach frequency. The Omnibus Rule extended direct liability under the HIPAA Security and Privacy Rules to BAs and required BA agreements that specify safeguards for PHI. The absence of a detectable shift in BA-involved breach frequency, however, suggests that extending legal liability alone may be insufficient to alter security outcomes [29]. Several explanations are plausible.

First, the rule primarily established accountability and notification obligations rather than prescriptive, enforceable security controls, leaving specific safeguards largely to individual organizations’ discretion. Second, BA agreements are contractual instruments whose existence does not guarantee that the underlying technical and administrative safeguards are implemented or maintained. Third, direct enforcement of the Security Rule against BAs has been limited relative to the size and growth of the third-party data-processing sector, thereby reducing the deterrent effect of the liability extension. Finally, the regulatory change preceded the increase in third-party risk identified in this study by several years, and it coincided with technological and threat-landscape changes that the 2013 rule was not designed to anticipate. Because this analysis observes only the realized trajectory, it cannot rule out the possibility that the Omnibus Rule constrained an increase that would otherwise have been steeper; the finding indicates no detectable reduction in observed involvement, rather than an absence of any regulatory influence.

These observations suggest that future regulatory efforts may need to move beyond liability assignment and breach notification toward more prescriptive and verifiable security requirements for BAs. Potential directions include mandatory minimum security controls or recognized certification for entities that process large volumes of PHI, requirements for data minimization and compartmentalization to limit aggregate exposure when a single vendor is compromised, and proactive oversight of high-volume BAs rather than reliance on postbreach notification. Because a single BA can aggregate data from many CEs, regulatory attention proportionate to the concentration of risk, rather than to entity type alone, may more effectively address the systemic third-party exposure documented in this analysis. These directions are consistent with international efforts to strengthen and harmonize health care data protection frameworks, which increasingly emphasize prescriptive safeguards, governance structures, and the integration of advanced security technologies [30].

### Limitations

This study has several limitations. First, the HHS OCR breach portal includes only breaches affecting 500 or more individuals and therefore excludes smaller incidents, which occur frequently and may have a substantial cumulative impact.

Second, the data are self-reported by CEs and BAs, and reporting practices may have changed over the study period. Increased regulatory attention, greater breach-detection capability, and evolving notification norms may have raised the likelihood that breaches were identified and reported in later years; some portion of the observed temporal increase, including in BA-involved breaches, may therefore reflect changes in reporting behavior rather than changes in the underlying incidence of breaches [31]. More specifically, the growth of hacking and ransomware, which are more readily detected and more consistently reported than earlier physical-media incidents, may have amplified the apparent shift in breach mechanisms independent of any true change in their relative frequency. Additionally, the 2013 Omnibus Rule altered which entities were required to report and how BA involvement was recorded, so part of the observed trend in BA involvement may reflect the evolution of the reporting framework itself rather than a change in underlying risk. Because these mechanisms operate in different directions, with improved detection inflating later counts while the reporting threshold and posting lags suppress them, the net effect on the observed trend cannot be determined. Additionally, breach type, breach location, and BA involvement are self-classified by the reporting entity at the time of submission, and inconsistent or imprecise categorization may introduce misclassification; for example, a breach involving multiple systems may be recorded under a single location category, which could affect the observed distribution of breach locations.

Third, the breach portal is subject to reporting and public-posting lags, particularly in the most recent year; breaches occurring late in the study window may not yet have been reported or posted at the time of data extraction, so the 2025 figures are provisional and likely understate that year’s true incident count.

Fourth, the BA-involvement flag does not distinguish between BAs that were directly responsible for a breach and those that were otherwise involved. Although the dataset distinguishes breaches filed by a BA from those filed by a CE that identified BA-involvement pathway-specific temporal trends were not examined in this study. The trends in this study describe BA involvement as a single combined measure, without considering the extent to which the 2 reporting pathways followed similar trajectories.

Fifth, the dataset does not include organizational characteristics such as size, revenue, or specific security controls, limiting the ability to adjust for potential confounders. Sixth, the temporal analysis identifies associations but cannot establish causation; the observed increase in BA involvement may reflect changes in reporting, definitional changes, secular trends, or genuine changes in risk.

Finally, the dataset lacks detailed information on the types of BA services involved, precluding analysis of which BA functions carry the greatest risk.

### Future Research

Future research should investigate the severity and impact of breaches involving BAs by using multivariate models that account for breach type, location, and entity characteristics. Subsequent studies should also examine whether the Omnibus Rule is effective at reducing the severity of breaches rather than their frequency. This could be done using interrupted time-series or quasi-experimental designs. Knowing which types of BA services are most at risk of breaches would help regulators and organizations make targeted changes. Finally, using quantile regression on BA breach severity data could reveal whether BA involvement has different effects across the severity distribution. Future work should also evaluate which technical and organizational safeguards most effectively reduce the risk of BA-related breaches, building on the growing body of research examining mitigation technologies such as encryption, access controls, and blockchain-based approaches [32]. Future work should also examine whether temporal trends differ between breaches filed by BAs and those filed by CEs that identify BA involvement, since the two reporting pathways may reflect different aspects of third-party risk.

### Conclusions

Building on prior evidence linking BA involvement to breach severity, this study traced how that third-party risk evolved across the full reporting era. BA-involved health care data breaches accelerated substantially, with the steepest increase occurring after 2020. Breaches involving BAs are more likely to occur through hacking and to involve network servers. They also impact more individuals than the number of incidents would suggest. The implementation of the 2013 Omnibus Rule’s extension of liability to BAs was not followed by a detectable reduction in reported BA-involvement rates. Instead, the increase beginning in 2020 coincided with the COVID-19 pandemic and the ransomware epidemic. Professionals in health information management and cybersecurity should establish proactive third-party risk management programs to address the growing concentration of breach risk in BA relationships.

## Abbreviations

BA: business associate
CE: covered entity
EMR: electronic medical record
HHS: Department of Health and Human Services
HIPAA: Health Insurance Portability and Accountability Act
HITECH: Health Information Technology for Economic and Clinical Health
OCR: Office for Civil Rights
OR: odds ratio
PHI: protected health information

## References

[1] (2024). Health Insurance Portability and Accountability Act of 1996 (HIPAA). Centers for Disease Control and Prevention.

[2] (2026). Summary of the HIPAA security rule. US Department of Health and Human Services.

[3] (2024). Breach portal: notice to the secretary of HHS breach of unsecured protected health information. US Department of Health and Human Services, Office for Civil Rights.

[4] Seh AH, Zarour M, Alenezi M (2020). Healthcare data breaches: insights and implications. Healthcare (Basel).

[5] Lee I (2022). An analysis of data breaches in the U.S. healthcare industry: diversity, trends, and risk profiling. Inf Secur J A Glob Perspect.

[6] Angst CM, Block ES, D’Arcy J, Kelley K (2017). When do IT security investments matter? Accounting for the influence of institutional factors in the context of healthcare data breaches. MIS Q.

[7] Coventry L, Branley D (2018). Cybersecurity in healthcare: a narrative review of trends, threats and ways forward. Maturitas.

[8] (2013). Modifications to the HIPAA privacy, security, enforcement, and breach notification rules under the Health Information Technology for Economic and Clinical Health Act and the Genetic Information Nondiscrimination Act; other modifications to the HIPAA rules. https://www.govinfo.gov/content/pkg/FR-2013-01-25/pdf/2013-01073.pdf.

[9] Moore W, Frye S (2019). Review of HIPAA, part 1: history, protected health information, and privacy and security rules. J Nucl Med Technol.

[10] Blumenthal D, Tavenner M (2010). The “meaningful use” regulation for electronic health records. N Engl J Med.

[11] Cresswell K, Domínguez Hernández A, Williams R, Sheikh A (2022). Key challenges and opportunities for cloud technology in health care: semistructured interview study. JMIR Hum Factors.

[12] Ignatovski M (2022). Healthcare breaches during COVID-19: the effect of the healthcare entity type on the number of impacted individuals. Perspect Health Inf Manag.

[13] Tebeje TH, Klein J (2021). Applications of e-health to support person-centered health care at the time of COVID-19 pandemic. Telemed J E Health.

[14] Ewoh P, Vartiainen T (2024). Vulnerability to cyberattacks and sociotechnical solutions for health care systems: systematic review. J Med Internet Res.

[15] He Y, Aliyu A, Evans M, Luo C (2021). Health care cybersecurity challenges and solutions under the climate of COVID-19: scoping review. J Med Internet Res.

[16] Gabriel MH, Noblin A, Rutherford A, Walden A, Cortelyou-Ward K (2018). Data breach locations, types, and associated characteristics among US hospitals. Am J Manag Care.

[17] Dolezel D, McLeod A (2019). Cyber-analytics: identifying discriminants of data breaches. Perspect Health Inf Manag.

[18] McLeod A, Dolezel D (2018). Cyber-analytics: modeling factors associated with healthcare data breaches. Decis Support Syst.

[19] Ignatovski M (2021). Contributing factors to the number of individuals impacted by data breaches in healthcare organizations [dissertation]. https://media.proquest.com/media/hms/PFT/2/oqlKM?_s=iJIAwK5edfJFWVFYLvZeIbrqYG0%3D.

[20] Ignatovski M (2024). For-profit versus non-profit cybersecurity posture: breach types and locations in healthcare organisations. Health Inf Manag.

[21] Abraham C, Chatterjee D, Sims RR (2019). Muddling through cybersecurity: insights from the U.S. healthcare industry. Bus Horiz.

[22] Krzyzanowski B, Manson SM (2022). Twenty years of the Health Insurance Portability and Accountability Act safe harbor provision: unsolved challenges and ways forward. JMIR Med Inform.

[23] Riek M, Böhme R (2018). The costs of consumer-facing cybercrime: an empirical exploration of measurement issues and estimates. Journal of Cybersecurity.

[24] Jiang JX, Ross JS, Bai G (2025). Ransomware attacks and data breaches in US health care systems. JAMA Netw Open.

[25] Cornejo GM (2026). Third-party risk in U.S. health care ransomware incidents: business associate involvement and breach size. Health Technol.

[26] Raghupathi W, Raghupathi V, Saharia A (2023). Analyzing health data breaches: a visual analytics approach. AppliedMath.

[27] (2018). Framework for improving critical infrastructure cybersecurity: version 1.1. https://www.nist.gov/publications/framework-improving-critical-infrastructure-cybersecurity-version-11.

[28] (2023). HITRUST.

[29] Subramanian H, Sengupta A, Xu Y (2024). Patient health record protection beyond the Health Insurance Portability and Accountability Act: mixed methods study. J Med Internet Res.

[30] Conduah AK, Ofoe S, Siaw-Marfo D (2025). Data privacy in healthcare: global challenges and solutions. Digit Health.

[31] (2022). Electronic health information: HHS needs to improve communications for breach reporting. https://www.gao.gov/products/gao-22-105425.

[32] Nemec Zlatolas L, Welzer T, Lhotska L (2024). Data breaches in healthcare: security mechanisms for attack mitigation. Cluster Comput.

